# Tezepelumab treatment for allergic bronchopulmonary aspergillosis

**DOI:** 10.1002/rcr2.1147

**Published:** 2023-04-17

**Authors:** Hiroaki Ogata, Kachi Sha, Yasuaki Kotetsu, Aimi Enokizu‐Ogawa, Katsuyuki Katahira, Akiko Ishimatsu, Kazuhito Taguchi, Atsushi Moriwaki, Makoto Yoshida

**Affiliations:** ^1^ Department of Respiratory Medicine National Hospital Organization Fukuoka National Hospital Fukuoka Japan

**Keywords:** allergic bronchopulmonary aspergillosis, asthma, mepolizumab, mucoid impaction, tezepelumab

## Abstract

An 82‐year‐old man had been diagnosed with asthma. He experienced repeated exacerbations requiring treatment with a systemic corticosteroid despite being treated with medications including high‐dose fluticasone furoate/umeclidinium/vilanterol, montelukast sodium, and theophylline; treatment with mepolizumab was then initiated. The patient had been free from exacerbations for 15 months; however, he suffered from post‐obstructive pneumonia and atelectasis secondary to mucoid impaction in the right middle lobe of the lung, accompanied by a productive cough, wheezing, dyspnea, and right chest pain. In addition to the development of mucus plugs, the levels of serum IgE specific to Aspergillus spp. became positive; a definite diagnosis of allergic bronchopulmonary aspergillosis (ABPA) was established. The patient underwent treatment with tezepelumab. Over 3 months, the mucus plugs and pulmonary opacities diminished gradually in parallel with the improvement in the control of asthmatic symptoms. Tezepelumab might provide a novel steroid‐sparing strategy for the management of ABPA, although further studies are required.

## Graphical abstract

The patient with allergic bronchopulmonary aspergillosis (ABPA) underwent treatment with tezepelumab; the mucus plugs and pulmonary opacities diminished gradually parallel to the improvement in the control of asthmatic symptoms. Tezepelumab might provide a novel steroid‐sparing strategy for the management of ABPA.

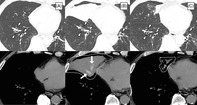

## INTRODUCTION

Allergic bronchopulmonary aspergillosis (ABPA) is an eosinophilic pulmonary disease caused by hypersensitivity to *Aspergillus* spp., frequently manifesting as severe asthma with mucus plugs and increased levels of blood eosinophil and serum immunoglobulin (Ig) E.^1^ Although it is well known that the standard treatment for ABPA is the administration of a systemic corticosteroid, this treatment is sometimes avoided due to its various and serious adverse effects, including a rise in the risk of infection, osteoporosis, and hyperglycemia.^2^ Thus, alternative therapeutic options for ABPA are in high demand.

Tezepelumab is a human IgG2 monoclonal antibody that binds specifically to thymic stromal lymphopoietin (TSLP), preventing its interaction with its heterodimeric receptor complex. Tezepelumab was demonstrated to improve the control of severe asthma by normalizing broad inflammatory pathways.^3^ It is therefore assumed to have a potential role in the management of ABPA; however, there has been no clinical report showing the effect of tezepelumab on ABPA. We now present a case of ABPA, developed during mepolizumab treatment, successfully treated with tezepelumab.

## CASE REPORT

**FIGURE 1:**
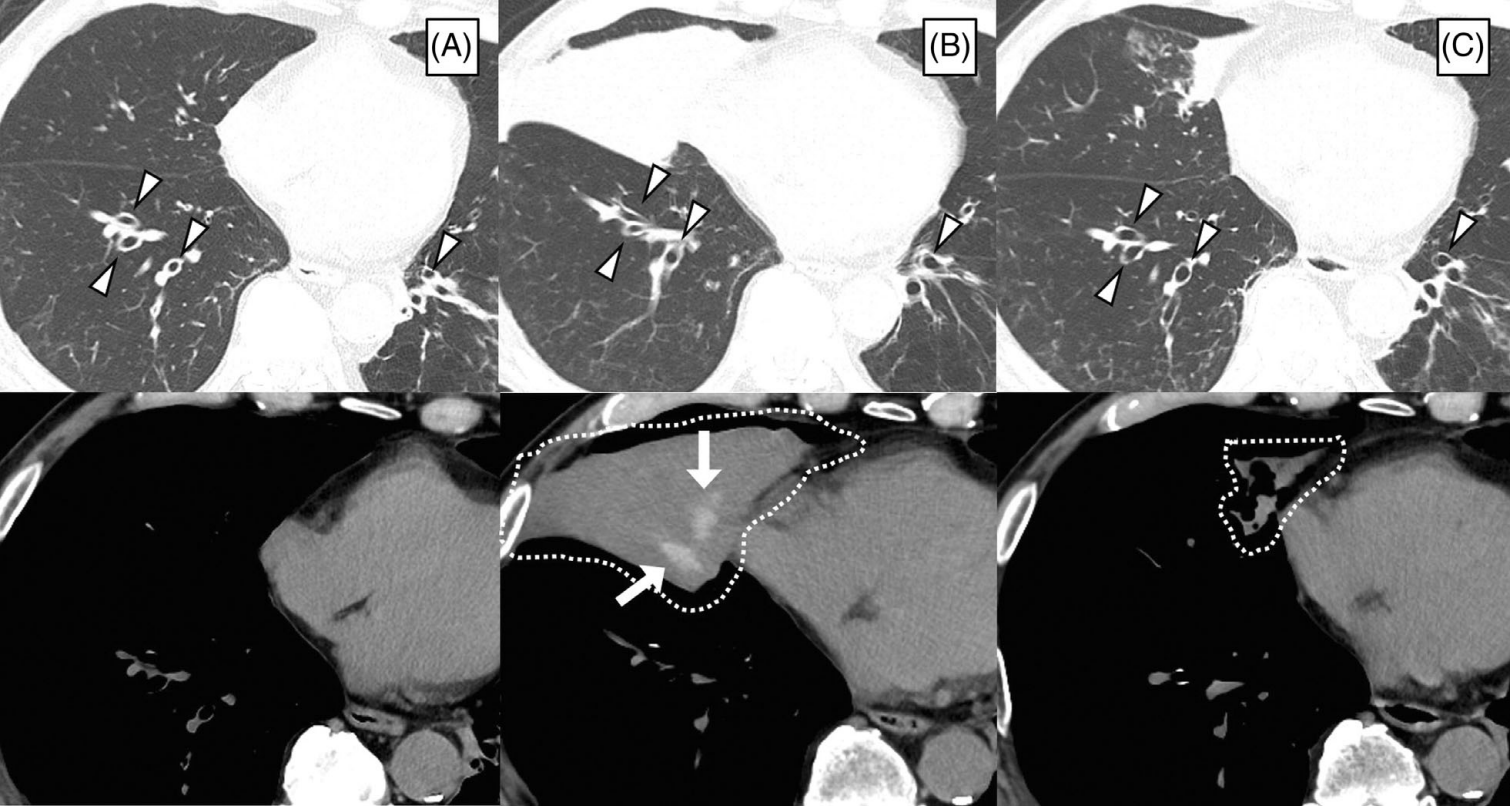
Computed tomography images of the chest (A) before the administration of mepolizumab, (B) when developing allergic bronchopulmonary aspergillosis during mepolizumab treatment, and (C) 3 months after initiating treatment with tezepelumab. Arrowheads indicate central bronchiectasis. High‐attenuation mucus plugs in the right middle lobe of the lung (white arrows), accompanied by atelectasis (dotted lines), were attenuated after the initiation of tezepelumab therapy

An 82‐year‐old Japanese man with a smoking history of 40 pack‐years, pulmonary emphysema, and bronchiectasis with colonization by *Pseudomonas aeruginosa* had been diagnosed with asthma and prescribed high‐dose fluticasone furoate/umeclidinium/vilanterol, montelukast sodium, and theophylline for years. However, the patient was prone to exacerbations of asthma requiring treatment with a systemic corticosteroid (three times per year). The peripheral blood test showed eosinophilia (560/μL), markedly elevated levels of total IgE (2153 IU/mL), and positive levels of IgE specific to *Aspergillus* spp. (2.83 IUA/mL). The computed tomography scans revealed central bronchiectasis, but there was no evidence of mucoid impaction (Figure 1A). Treatment with mepolizumab was started, and the patient had been free from exacerbations requiring systemic steroid use for a while; however, 15 months after the initiation of mepolizumab, he suffered from post‐obstructive pneumonia and atelectasis secondary to high‐attenuation mucus in the right middle lobe of the lung (Figure 1B), accompanied by a productive cough, wheezing, dyspnea, and right chest pain. In addition to the development of mucus plugs, the levels of serum IgE specific to Aspergillus spp. were positive; a definite diagnosis of ABPA was established, although blood eosinophilia was still suppressed, and the sputum culture was not positive for *Aspergillus* spp. His pneumonia was treated with an intravenous infusion of sulbactam/cefoperazone, whereas a systemic corticosteroid was not used to treat the exacerbation of asthmatic symptoms in order to prevent treatment failure in antibiotic therapy. To avoid a relapse of pneumonia and the progression of *P. aeruginosa* infection, a systemic steroid was still not used after pneumonia improvement; instead, the patient underwent treatment with tezepelumab. The mucus plugs and pulmonary opacities diminished gradually over 3 months (Figure 1C) in parallel with improvement in the control of asthmatic symptoms; the levels of total IgE decreased from 1426 to 722 IU/mL. A systemic steroid has not been prescribed for the patient since treatment with tezepelumab was initiated.

## DISCUSSION

Although a systemic corticosteroid plays a pivotal role in the treatment of ABPA, it is sometimes avoided because of its various severe side effects;^2^ a steroid‐sparing strategy for the management of ABPA is needed. Recent reports have demonstrated the efficacy of mepolizumab, a monoclonal antibody against interleukin (IL)‐5, in the treatment of ABPA; however, there have also been several case reports of treatment failure with mepolizumab for mucoid impaction in ABPA.^4^ As far as we are aware, the present case is the first to report the improvement of ABPA after switching from mepolizumab to tezepelumab.

With regard to the pathogenesis of ABPA, not only IL‐5‐driven airway eosinophilia but also IL‐13‐induced mucus hypersecretion plays a crucial role in the development of mucoid impaction. Moreover, group 2 innate lymphoid cells (ILC2s) produce IL‐5 and IL‐13, promoting disease progression in ABPA. Therefore, tezepelumab can be a suitable agent for the treatment of ABPA, since it binds to and inhibits TSLP, an epithelial cell–derived proinflammatory cytokine implicated in multiple downstream processes including IL‐5, IL‐13 and ILC2 pathways.

An anti‐IL‐5 antibody, mepolizumab and an anti‐IL‐5 receptor antibody, benralizumab, have been reported to improve the condition of ABPA by diminishing IL‐5‐mediated eosinophilic airway inflammation. However, as in the present case, these anti‐IL‐5 biologics sometimes do not contribute substantially to preventing the development of mucus plugs in ABPA.^4^ This may be because non‐eosinophilic or IL‐5‐independent pathways are involved in the mechanisms underlying mucus development. On the other hand, dupilumab, an anti‐IL‐4/IL‐13 monoclonal agent, is expected to prevent and reduce mucus plug formation, developed principally via the upregulated IL‐13 pathway. However, it poses the risk of exacerbating eosinophilic inflammation, since it is known to initially induce or worsen hypereosinophilia,^5^ unlike tezepelumab.^3^ Hence, among biologics for asthma, tezepelumab is speculated to have an advantage for patients with ABPA.

In the present case, peripheral eosinophilia had been suppressed throughout the mepolizumab treatment, even when mucoid impaction appeared. Taking this into account, the attenuation of the effects of mepolizumab on ABPA was probably not due to the generation of neutralizing antibodies against mepolizumab. Thus, switching from mepolizumab to benralizumab, another anti‐IL‐5 therapy, might have been ineffective for the present patient.

Antifungal therapy, another treatment option for ABPA, was not used in the present case. However, antifungal agents are known to play only an adjunctive role in the management of ABPA; they should be prescribed not alone but with a systemic corticosteroid.^6^ Thus, there is a lack of evidence to support the use of antifungals as a steroid‐sparing medicine.

Some limitations should be noted. First, the measurements of fractional exhaled nitric oxide or pulmonary function were not accessible in the present case because of the pandemic coronavirus disease 2019. Second, the number or percentage of eosinophils in sputum was not examined. Lastly, the long‐term efficacy and safety of tezepelumab for ABPA remains unclear. The influence of long‐term use of tezepelumab on *P. aeruginosa* colonization is also unknown. Therefore, careful monitoring of the patient is ongoing.

In conclusion, tezepelumab might provide a novel therapeutic approach for managing ABPA. Since tezepelumab is a potential ‘game changer’ in the management of severe and difficult‐to‐treat asthma, it can be a promising alternative to systemic steroid use in patients with ABPA. Further studies evaluating the efficacy and safety of tezepelumab against ABPA are highly warranted.

## AUTHOR CONTRIBUTIONS

Hiroaki Ogata contributed to the conception of the work, the acquisition and interpretation of data for the work, and drafting of the manuscript. Yasuaki Kotetsu, Katsuyuki Katahira, Aimi Enokizu‐Ogawa, Kazuhito Taguchi, Akiko Ishimatsu, Kazuhito Taguchi, and Atsushi Moriwaki contributed to the interpretation of data for the work and revision of the manuscript. Makoto Yoshida contributed to the conception of the work, interpretation of the data, and revision of the manuscript. All authors critically reviewed the manuscript and approved the final version.

## CONFLICT OF INTEREST STATEMENT

None declared.

## ETHICS STATEMENT

The authors declare that appropriate written informed consent was obtained for the publication of this manuscript and accompanying images.

## Data Availability

The data that support the findings of this study are available from the corresponding author upon reasonable request.
